# Coronary microcirculation damage in anthracycline cardiotoxicity

**DOI:** 10.1093/cvr/cvab053

**Published:** 2021-02-19

**Authors:** Carlos Galán-Arriola, Jean Paul Vílchez-Tschischke, Manuel Lobo, Gonzalo J López, Antonio de Molina-Iracheta, Claudia Pérez-Martínez, Rocio Villena-Gutiérrez, Álvaro Macías, Iván A Díaz-Rengifo, Eduardo Oliver, Valentin Fuster, Javier Sánchez-González, Borja Ibanez

**Affiliations:** 1 Centro Nacional de Investigaciones Cardiovasculares (CNIC), c/Melchor Fernández Almagro 3, 28029 Madrid, Spain; 2 Centro de Investigación Biomédica en Red en Enfermedades Cardiovasculares (CIBERCV), c/Melchor Fernández Almagro 3, 28029 Madrid, Spain; 3 Complejo Hospitalario Ruber Juan Bravo, c/Juan Bravo 29, 28006 Madrid, Spain; 4 Facultad de Veterinaria de León, Campus de Vegazana s/n, 24007 León, Spain; 5 Icahn School of Medicine at Mount Sinai, The Zena and Michael A. Wiener Cardiovascular Institute, 1 Gustave L. Levy Place, New York, NY 10029-6574, USA; 6 Philips Healthcare, c/María de Portugal 1, 28050 Madrid, Spain; 7 Department of Cardiology, IIS-Fundación Jiménez Díaz Hospital, Av. de los Reyes Católicos 2, 28040 Madrid, Spain

**Keywords:** Cardiotoxicity, Anthracyclines, Cardio-oncology, Cardiac perfusion, Coronary physiology, Microcirculation

## Abstract

**Aims:**

The aim of this study was to study changes in coronary microcirculation status during and after several cycles of anthracycline treatment.

**Methods and results:**

Large-white male pigs (*n*=40) were included in different experimental protocols (ExPr.) according to anthracycline cumulative exposure [0.45 mg/kg intracoronary (IC) doxorubicin per injection] and follow-up: control (no doxorubicin); single injection and sacrifice either at 48 h (ExPr. 1) or 2 weeks (ExPr. 2); 3 injections 2 weeks apart (low cumulative dose) and sacrifice either 2 weeks (ExPr. 3) or 12 weeks (ExPr. 4) after third injection; five injections 2 weeks apart (high cumulative dose) and sacrifice 8 weeks after fifth injection (ExPr. 5). All groups were assessed by serial cardiac magnetic resonance (CMR) to quantify perfusion and invasive measurement of coronary flow reserve (CFR). At the end of each protocol, animals were sacrificed for *ex vivo* analyses. Vascular function was further evaluated by myography in explanted coronary arteries of pigs undergoing ExPr. 3 and controls. A single doxorubicin injection had no impact on microcirculation status, excluding a direct chemical toxicity. A series of five fortnightly doxorubicin injections (high cumulative dose) triggered a progressive decline in microcirculation status, evidenced by reduced CMR-based myocardial perfusion and CFR-measured impaired functional microcirculation. In the high cumulative dose regime (ExPr. 5), microcirculation changes appeared long before any contractile defect became apparent. Low cumulative doxorubicin dose (three bi-weekly injections) was not associated with any contractile defect across long-term follow-up, but provoked persistent microcirculation damage, evident soon after third dose injection. Histological and myograph evaluations confirmed structural damage to arteries of all calibres even in animals undergoing low cumulative dose regimes. Conversely, arteriole damage and capillary bed alteration occurred only after high cumulative dose regime.

**Conclusion:**

Serial *in vivo* evaluations of microcirculation status using state-of-the-art CMR and invasive CFR show that anthracyclines treatment is associated with progressive and irreversible damage to the microcirculation. This long-persisting damage is present even in low cumulative dose regimes, which are not associated with cardiac contractile deficits. Microcirculation damage might explain some of the increased incidence of cardiovascular events in cancer survivors who received anthracyclines without showing cardiac contractile defects.

## Graphical Abstract

**Figure:**
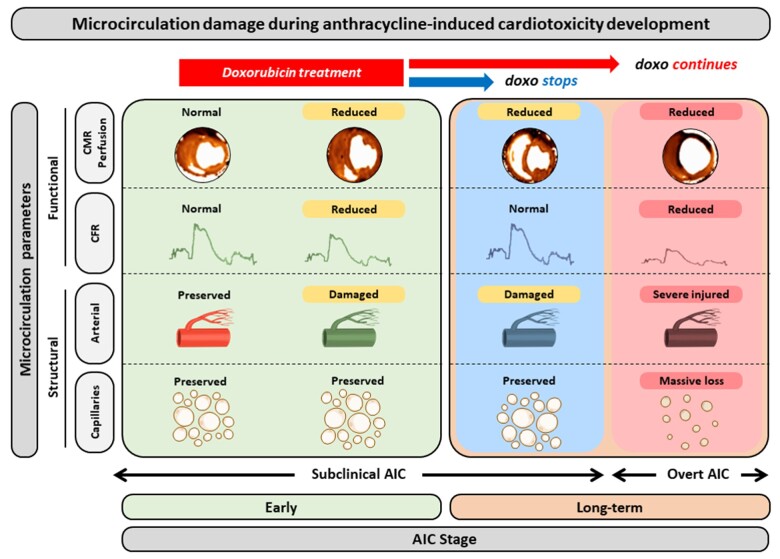

**Time for primary review: 20 days**

## 1. Introduction

Cardiotoxicity is a well-known side effect of cancer treatments and contributes to the elevated cardiovascular mortality rate among cancer survivors.^1^^,^^2^ The wide spectrum of cardiotoxicity presentations after cancer therapy includes myocardial ischaemia, hypertension, left ventricular (LV) systolic dysfunction, myocarditis and pericarditis, and microvascular damage. Damage to the coronary microcirculation is a well-known cardiotoxic side effect of radiotherapy;^2^ however, the effect of other anti-cancer agents on the coronary microvasculature is less well described. Anthracyclines are among the most widely prescribed chemotherapy drugs, but there is little published evidence that their cytotoxic effects include damage to the coronary microcirculation. *In vitro*, the anthracycline drug doxorubicin has been shown to induce injury to endothelial cells, seemingly through a process mediated by reactive oxygen species (ROS) production.^3–5^ Moreover, doxorubicin induces apoptosis^6^ and a premature senescent phenotype^7^ in cultured smooth muscle cells. The effect of doxorubicin on the cardiac vascular compartment has been studied in small animals (mice and rats), but only by histopathology after sacrifice.^8^^,^^9^ Observational studies in chemotherapy patients have suggested that anthracyclines might reduce myocardial perfusion without affecting LV systolic dysfunction.^10^ Although there is indirect evidence suggesting a cardiotoxic effect of anthracyclines on the myocardial vasculature, there is a lack of a comprehensive prospective studies evaluating the microcirculation from anatomical and functional perspectives across all stages of cardiotoxicity, from pre-exposure through subclinical effects to overt cardiac dysfunction.

We recently described a pig model of anthracycline-induced cardiotoxicity in which cardiac dysfunction is seen after five fortnightly intracoronary (IC) doxorubicin injections.^11^ This is an ideal model for addressing the question of anthracycline-induced cardiotoxicity because it allows serial study across cardiotoxicity stages: the healthy heart (pre-exposure), subclinical cardiotoxicity with no contractile defect (no LV function alterations), and advanced stages with overt LV systolic depression. The effects of low cumulative doses vs. high cumulative doses can also be studied by this approach. In addition, the use of large animals makes the model amenable to the use of clinical tools for the *in vivo* evaluation coronary physiology (IC flow-pressure wires) and quantitative perfusion (by cardiac magnetic resonance, CMR). Additional information can be obtained *ex vivo* at the end of follow-up. In this study, we have studied the evolution of microcirculation status across all stages of the pig anthracycline-induced cardiotoxicity model.

## 2. Methods

### 2.1 Study design

The study was approved by the Institutional Animal Research Committee (PROEX074/15) and conducted in accordance with recommendations of the Guide for the Care and Use of Laboratory Animals (Directive 2010/63/EU).

**Figure 1:**
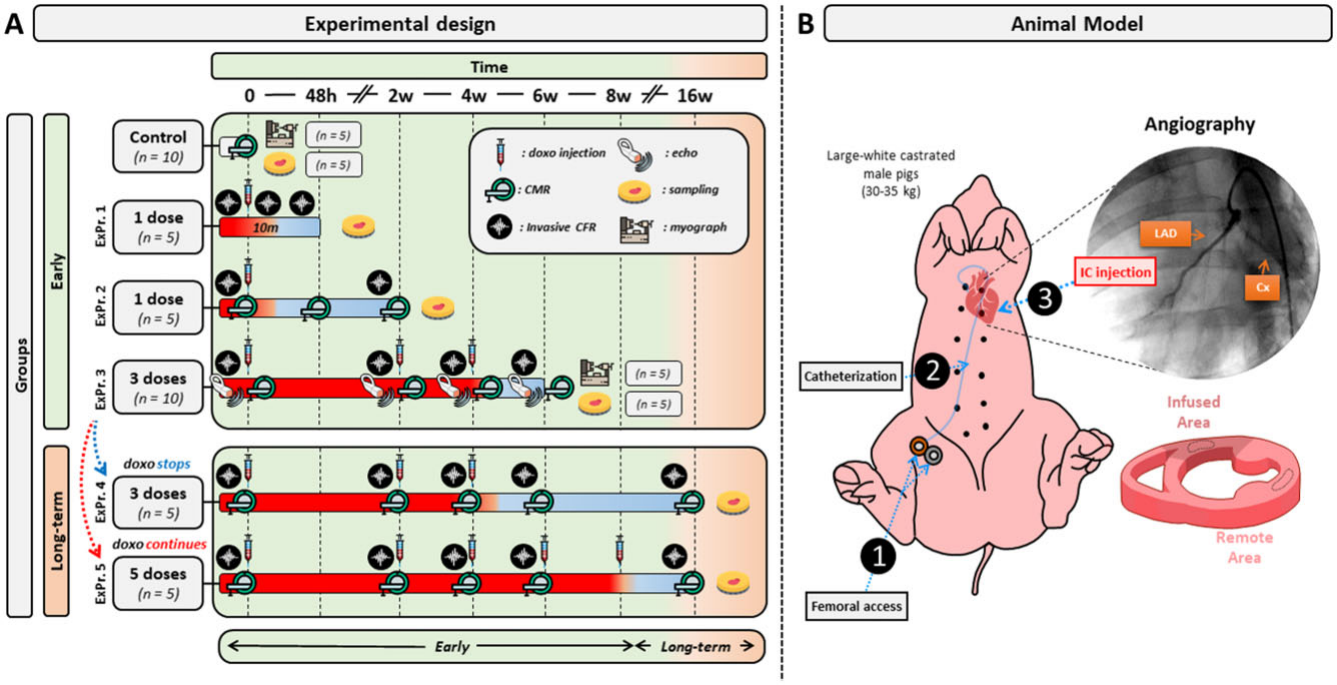
Study design and animal model. Large-white castrated male pigs weighing 30–35 kg were used in this study. Animals were divided into six groups. A *Control group* of untreated animals (*n*=10), *ExPr.1 group*: pigs received 1 IC doxorubicin sacrificed 48 h later (*n*=5). *ExPr.2 group*: pigs received 1 IC doxorubicin sacrificed 2 weeks later (*n*=5), *ExPr.3 group*: pigs received three IC doxorubicin doses followed by 2 dose-free weeks before sacrifice (*n*=10). *ExPr.4 group*: pigs received 3 bi-weekly IC doxorubicin doses followed by 12 dose-free weeks (*n*=5). *ExPr.5 group*: animals received five bi-weekly IC doxorubicin doses followed by eight dose-free weeks (*n*=5). CMR scans and coronary physiology assessment were performed before doxorubicin infusion. Infused and remote myocardial areas were collected at sacrifice for further analysis. Animals in the Expr.3 (only 5), 4, and 5 are the same that were included in a previous publication, where LVEF was already reported.^11^

The study design is summarized in *Figure 1*. A total of 40 male large-white pigs (30–35 kg) were included in different experimental protocols (ExPr.). Controls were healthy pigs with no doxorubicin exposure (*N*=10, 5 for the CMR and CRF studies, and 5 for the myography studies); single injection and sacrifice 48 h later (ExPr. 1, *N*=5); single injection and sacrifice 2 weeks later (ExPr. 2, *N*=5); three injections 2 weeks apart (low cumulative dose) and sacrifice 2 weeks after third injection (ExPr. 3, *N*=10, 5 for the CMR and CRF studies, and 5 for the myography studies); 3 injections 2 weeks apart (low cumulative dose) and sacrifice at 16 weeks follow-up (i.e. 12 weeks after third injection) (ExPr. 4, *N*=5); 5 injections 2 weeks apart (high cumulative dose) and sacrifice at 16 weeks follow-up (i.e. 8 weeks after fifth injection) (ExPr. 5, *N*=5).

A total of 15 pigs (5 pigs from ExPr. 3–5) were the same as those included in a previous report describing the model.^11^ The other 25 pigs were specifically included for this study.

Coronary flow reserve (CFR) was determined by serial invasive coronary physiology studies with flow-pressure combo wires at the time points indicated in *Figure 1*, before IC doxorubicin injections. In ExPr. 1, CFR was also evaluated 10 min after the IC doxorubicin injection. Likewise, perfusion was also quantified at the time points indicated in *Figure 1* using a serial non-invasive CMR protocol described in a previous report.^12^

**Figure 2:**
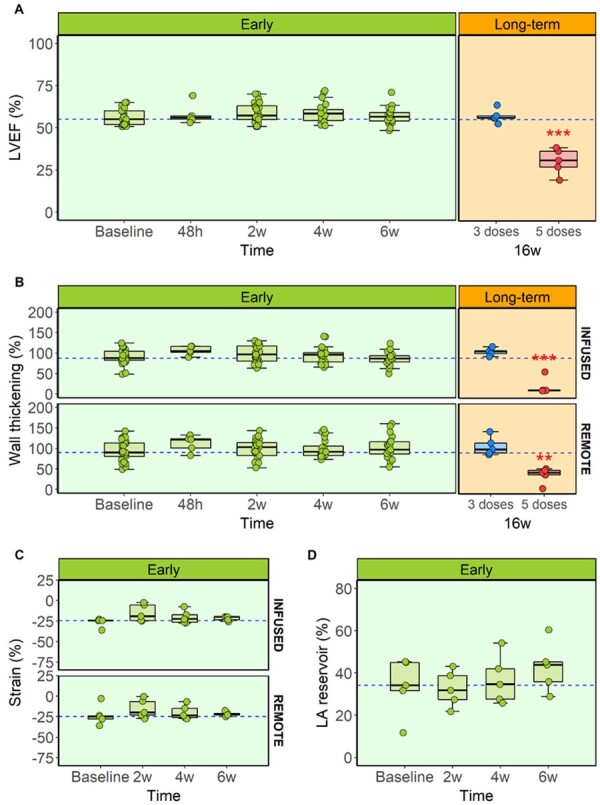
Global and regional cardiac function assessment during anthracycline-induced cardiotoxicity development. (*A*) CMR-LVEF in early and long-term stages for all ExPr groups. (*B*) CMR-wall thickening analysis for the infused and remote myocardial areas in early and long-term stages for all ExPr groups. Sample size for panels (*A*) and (*B*): baseline=30, 48 h=10, 2w=25, 4w=20, 6w=20, 16w=5 each. (*C*) Circumferential myocardial strain by echocardiography in ExPr. 3 in infused and remote areas during early stages. (*D*) CMR-diastolic function evaluation by left atrium reservoir analysis in ExPr. 3 during early stages. Sample size for panels (*C*) and (*D*)=5. Asterisks indicate statistically significant differences compared with Baseline: *P<0.05, **P<0.01, ***P<0.001. For panels (*A*) and (*D*): one-way ANOVA was performed to detect differences between Baselines and the rest of the time points (paired, corrected by Bonferroni). For panels (*B*) and (*C*): two-way ANOVA was performed to detect differences between time points and infused-remote areas within the same time point (both paired, corrected by Bonferroni). Boxplots represent median and interquartile range, while whiskers show maximum and minimum values. Circles represent individual data.

### 2.2 Doxorubicin administration

Doxorubicin was administered as reported previously.^11^ Briefly, animals were anaesthetized (20 mg/kg of ketamine, 2 mg/kg of xylazine, and 0.5 mg/kg of midazolam by intramuscular injection) and maintained with intravenously perfusion of the same anaesthetics cocktail (per hour) they were also endotracheally intubated. Intramuscular buprenorphine (0.01 mg/kg) was also intramuscular administrated before the catheterism. The femoral artery was then accessed by the Seldinger technique, a 7-Fr sheath was inserted, and 150 UI/kg of intravenous heparin was administrated. A 5-Fr coronary catheter was inserted through the femoral sheath and placed at the origin of the left coronary artery. Under angiography guidance, a 0.014 mm coronary guidewire was positioned distally in the left anterior descending (LAD) coronary artery, and the catheter was docked in the proximal LAD. A 0.45 mg/kg dose of doxorubicin (Farmiblastina^®^, Pfizer) diluted in 30 mL saline was given as a slow bolus injection over 3 min, and a coronary angiography was recorded after infusion. Monitoring of electrocardiography and systemic blood pressure revealed no changes during doxorubicin infusion.

In animals receiving serial doxorubicin doses 2 weeks apart, the procedure was alternated between the right and left arterial femoral routes to reduce vascular complications.

### 2.3 Invasive measurement of coronary flow reserve

A coronary pressure-flow guidewire (VOLCANO Combowire**^®^**) was advanced through the guiding catheter and placed either distally in the LAD (to evaluate the doxorubicin-infused territory) or distally in the left circumflex coronary artery (LCx) (to evaluate the remote myocardium). CFR was assessed before each doxorubicin infusion and calculated as the ratio of basal to maximum hyperaemia. The hyperaemia protocol consisted of IC administration of 0.5 mg/kg papaverine to the LAD or LCx coronary arteries, as previously described.^12^

### 2.4 Coronary perfusion by CMR

All CMR exams were performed before doxorubicin administration, using a 3 Tesla Philips Achieva Tx whole-body scanner (Philips Healthcare, Best, the Netherlands) equipped with a 32-element phased-array cardiac coil. Imaging studies were performed under the same anaesthesia used for doxorubicin administration. Cardiac quantitative perfusion was estimated using dynamic acquisition with dual-saturation recovery (TS=20, 80 ms) during gadolinium-based contrast administration (0.1 mmol/kg), as previously described.^12^ After perfusion map generation, regions of interest were analysed in the infused and remote areas using dedicated software (MR Extended Work Space 2.6, Philips Healthcare).

### 2.5 Tissue sampling and *ex vivo* analysis

After the last programmed CMR exam (different in each study group; *Figure 1*), animals were sacrificed by intravenous injection of pentobarbital in overdose (50 mg/kg intravenously). The whole heart was excised, and samples from the doxorubicin-infused region (anteroseptal wall) and the remote area were collected for analysis by histology, immunostaining, and western blotting. For the histology and immunostaining, samples were fixed in 4% formalin for 1 week and then transferred to 70% ethanol. Samples were then embedded in paraffin and cut in to 4 μm sections for staining with haematoxylin and eosin, Masson trichrome, and Sirius Red. Stained sections were scanned and 20 × 20 and 40 × 40 magnification images were obtained of myocardial arteries and arteriole analyses.

### 2.6 Arterial damage analysis

The intima, media, and adventitia layers and perivascular collagen were assessed by two independent expert pathologists blinded to study group. Pathology was assessed in every artery in the slice measuring the external perimeter of the vessel, including arterioles and small, medium, and large arteries. Before taking measurements, arterial damage was graded according to a damage scale created specifically for this study (Supplementary material online, *Figure S1*). The damage-scale grades were as follows: Grade 0, no lesions; Grade 1, minimal tunica intima hyperplasia and no tunica media lesions; Grade 2, mild-to-moderate tunica intima hyperplasia featuring myofibroblast proliferation and extracellular matrix accumulation, accompanied by tunica media disorganization with only occasional loss of isolated smooth muscle cells; Grade 3, moderate tunica intima hyperplasia with occasional luminal stenosis, accompanied by tunica media degeneration with moderate loss of smooth muscle cells and their replacement with extracellular matrix or collagen; and Grade 4, moderate-to-severe tunica intima hyperplasia with marked luminal stenosis, as well as tunica media degeneration with severe loss of smooth muscle cells and their replacement with extracellular matrix or collagen.

### 2.7 Capillary bed evaluation

Capillary density (per mm^2^) was measured by macro-based analysis of CD31 immunohistochemistry in 10 × 10 magnification images using FIJI software (ImageJ). The macro operation is summarized in Supplementary material online, *Figure S2*.

### 2.8 Vascular function evaluation by myography

Five additional pigs undergoing ExPr. 3 and controls (*N*=5 each) were included in order to better study *ex vivo* vascular function in explanted coronary arteries from the infused region.

Once individuals were sacrificed (same method reported in tissue sampling section), hearts were completely excised, washed with saline and preserved in Krebs–Henseleit solution (KHS) (115 mM NaCl, 2.5 mM CaCl2, 4.6 mM KCl, 1.2 mM KH2PO4, 1.2 mM MgSO4, 25 mM NaHCO3, 11.1 mM glucose, and 0.01 mM EDTA). After that, the LAD coronary artery was carefully dissected and cleaned of cardiac tissue, and cut into ∼2 mm long segments at several levels (summarized in Supplementary material online, *Figure S3*). Coronary rings were then mounted on two tungsten wires in a wire myograph system (620M, Danish Myo Technology A/S, Hinnerup, Denmark) and immersed in 37°C KHS with constant gassing (95% O_2_ and 5% CO_2_). Wire myography was performed as previously described.^13^^,^^14^ Optimal vessel distension was determined by normalization using the Laplace Equation [Tension = (pressure × radius)/thickness] to calculate the position at which the tension was equivalent to an intraluminal pressure of 100 mmHg (L100);^13^^,^^14^ vessels were then set up to the optimal tension (physiological distension, 0.9 of L100). Finally, for each vessel segment, a linear regression was calculated between the diameter and tension. Vessel stiffness was compute as the slope from the linear regression from this graph.

After equilibration for 30 min, vasoconstriction was studied by exposing the coronary rings first to 120 mM KCl. Vasodilation was assessed by examining the response to increasing doses of papaverine (from 10 to 1 mM; master formula) or bradikynin (from 1 pM to 10 µM; Sigma-Aldrich) in segments previously contracted with acetylcholine 0.5 µM (Sigma-Aldrich). Drug treatments were separated by extensive washes and a stabilization period of at least 30 min.

### 2.9 Statistical analysis

According to the data distribution, continuous variables were calculated as mean ± SD or median and interquartile range (Q1–Q3). Data normality was assessed with the Saphiro–Wilk test. The *t-*test was used for two-group comparisons, and changes in each imaging variable were assessed by one-way repeated-measures analysis of variance (ANOVA). To identify time points showing statistically significant differences from baseline, pairwise paired *t-*tests were performed with *P*-value adjustment for multiple comparisons (Bonferroni method). For CFR and quantitative perfusion analysis in the grouped analysis of subclinical stages, only data from Groups 5 and 6 (*Cardiotoxic* and *Recovery*) were used because their values were complete for all time points and they shared the same doxorubicin regime until week 6. For infused vs. remote variables comparisons, two-way factorial paired ANOVA was used. In the pathology analysis of vessel injury, between-group differences in damage score were detected with a *χ*^2^ analysis between each experimental group and the control group as a *post-hoc* analysis.

For myograph experiments, logarithmic curves were created and differences between control and doxorubicin animal arteries subjected to several drugs were detected with ANOVA analysis comparing the two multinomial logistic models. Differences were considered statistically significant at *P*<0.05. All data were analysed with RStudio [RStudio Team (2015): Integrated Development for RStudio, Inc., Boston, MA, USA], and graphics were created with the ggplot2 package.

## 3. Results

Doxorubicin administration was not associated with adverse reactions during injection (no changes were noted in the ECG or systolic arterial pressure), and there were no casualties throughout the study. Mean arterial pressure and heart rate did not significantly changed across the weeks of doxorubicin treatment (Supplementary material online, *Figure S4*).

**Figure 3:**
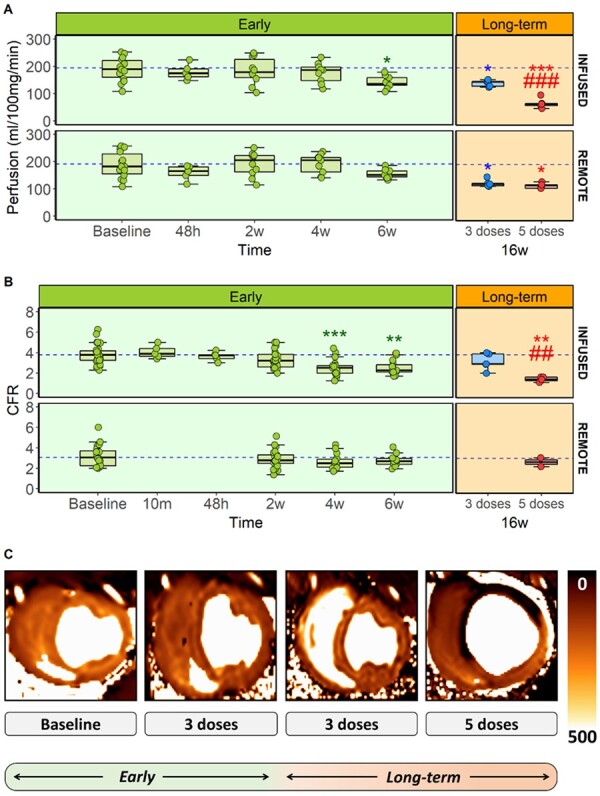
Cardiac microvascular function assessment during anthracycline-induced cardiotoxicity development. (*A*) CMR-quantitative perfusion for the infused and remote myocardial areas in early and long-term stages for all ExPr groups (sample size: baseline=14, 48 h=5, 2w=10, 4w=9, 6w=9, 16w=5 each). (*B*) CFR for the infused and remote myocardial areas in early and long-term stages for all ExPr groups (sample size for infused area: baseline=30, 10m=5, 48 h=5, 2w=25, 4w=19, 6w=15, 16w=5 each. Sample size for remote area: baseline=20, 10m=0, 48h=0, 2w=20, 4w=14, 6w=10, 16w=2 only for 5 doses). (*C*) Representative CMR-based cardiac perfusion maps for baseline, 3 doses at 6 and 16 weeks and 5 doses at 16 weeks groups. Asterisks indicate statistically significant differences compared with baseline: *P<0.05, **P<0.01, ***P<0.001. Hashes indicate statistically significant differences compared with respective remote: #*P*<0.05, ##*P*<0.01, ###*P*<0.001. Two-way ANOVA was performed to detect differences between time points and infused-remote areas within the same time point (both paired, corrected by Bonferroni). Boxplots represent median and interquartile range, while whiskers show maximum and minimum values. Circles represent individual data.

**Figure 4:**
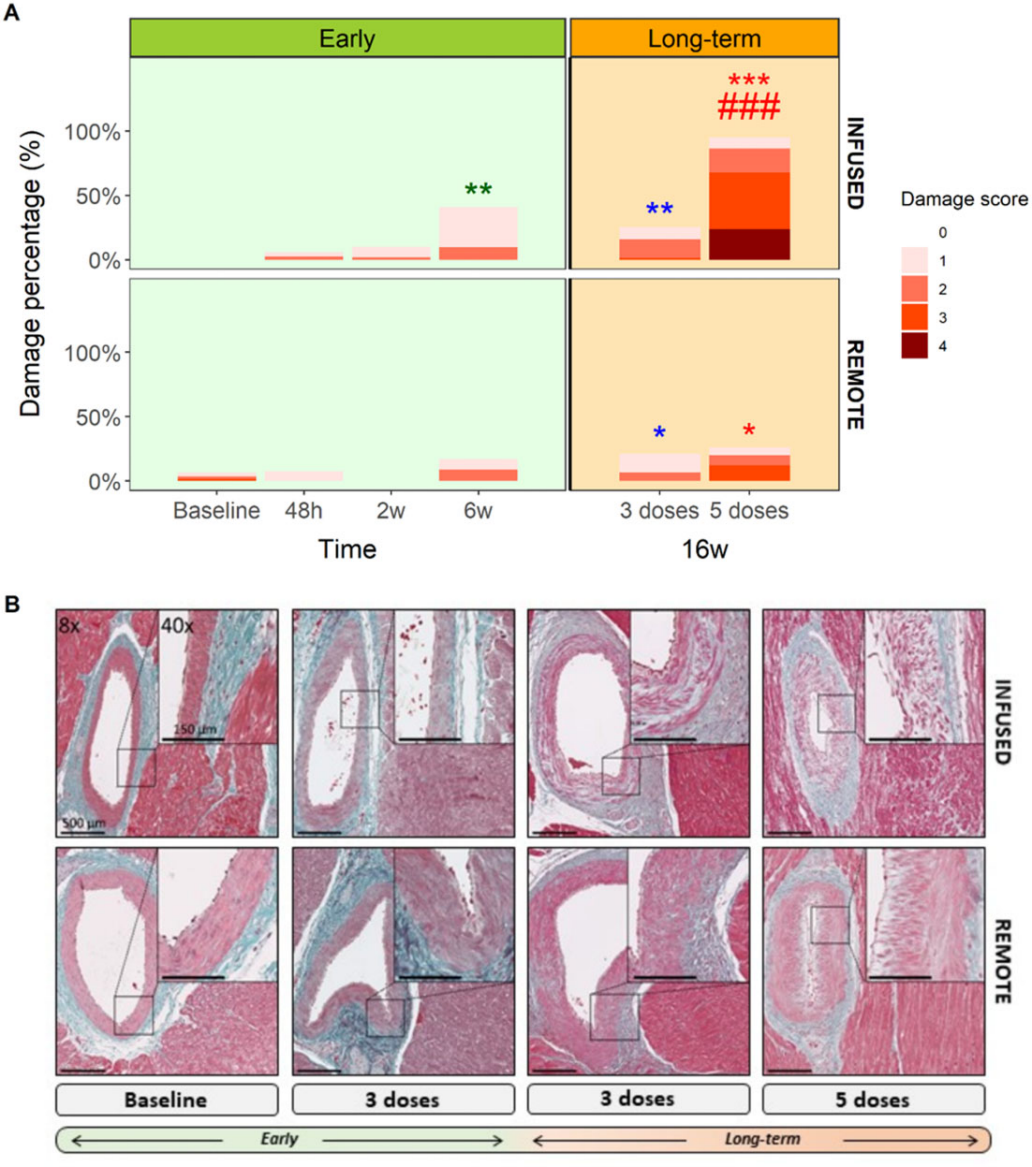
Pathology evaluation of microvasculature during anthracycline-induced cardiotoxicity development. (*A*) Stacked barplots indicating artery’s percentage evaluated with each damage score for each group and myocardial area (*n*=5 animals, ∼11 arteries scored per individual). (*B*) Representative arteries from infused and remote areas stained by Masson’s trichrome for baseline, 3 doses at 6 and 16 weeks and 5 doses at 16 weeks groups. Asterisks indicate statistically significant differences compared with baseline: *P<0.05, **P<0.01, ***P<0.001. Hashes indicate statistically significant differences compared with respective remote: #*P* <0.05, ##*P*<0.01, ###*P*<0.001. Chi-squared test were performed between to compare proportions with baseline and their respective remote area.

**Figure 5:**
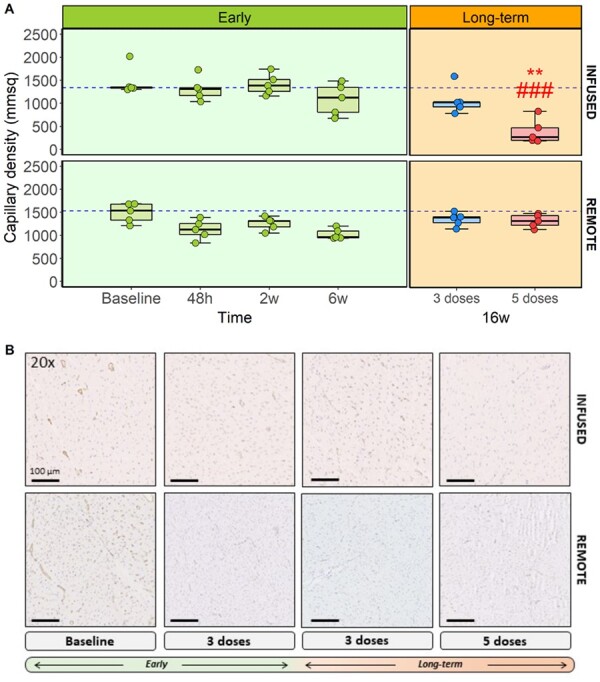
Capillary niche evaluation during anthracycline-induced cardiotoxicity development. (*A*) Capillary density for each group and myocardial area (*n*=5). (*B*) Asterisks indicate statistically significant differences compared with baseline: *P<0.05, **P<0.01, ***P<0.001. Hashes indicate statistically significant differences compared with respective remote: #*P*<0.05, ##*P*<0.01, ###*P*<0.001. Two-way ANOVA was performed to detect differences between baseline and the rest of the groups (unpaired); and between infused-remote areas within the same group (paired, corrected by Bonferroni). Boxplots represent median and interquartile range, while whiskers show maximum and minimum values. Circles represent individual data.

Global and regional cardiac contractile function (both in infused and remote regions) by CMR and echocardiography (strain) across are presented in *Figure 2*. CMR-determined quantitative perfusion and CFR data for the infused and remote regions across time are presented in *Figure 3*. Histology and immunohistochemistry data obtained at sacrifice at all different time points are presented in *Figures 4 and 5*.

### 3.1 Effect on the microvasculature of acute exposure to IC doxorubicin

We previously reported that IC doxorubicin does not provoke deterioration in LV systolic function until a certain cumulative dose is reached (after four fortnightly 0.45 mg/kg injections) (*Figure 2A*).^11^ The original description of the model did not include assessment of the status of the vasculature during the subclinical stage (i.e. after only three biweekly injections of doxorubicin, a moment where no global nor regional cardiac motion defects are observed) (*Figure 2*). To exclude acute toxicity due to the local administration route, here, we first studied the effects of a single IC doxorubicin injection. To this aim, we assessed the status of microcirculation anatomy (CMR-determined quantitative perfusion) and function (invasively determined CFR) before and after a single 0.45 mg/kg doxorubicin IC administration, as well as 48 h and 2 weeks later. Assessment at these later times points also included post-mortem histology analysis.

There were no changes in invasively determined CFR immediately after doxorubicin injection (*Figure 3B*). At 48 h after a single IC doxorubicin injection, infused region CMR-determined quantitative perfusion and invasively determined CFR were not different from baseline (199±40 vs. 180±29.3 mL/100 g/min for CMR-perfusion and 3.7±1.22 vs. 3.63±0.45 for CFR at baseline and 48 h, respectively). This was maintained at 2 weeks post-injection (199±40 vs. 183±49.2 mL/100 g/min for perfusion and 3.7±1.22 vs. 3.57±0.9 for CFR at baseline and 2 weeks, respectively) (*Figure 3*). CMR-quantitative perfusion and invasive CFR were also determined at the remote myocardium (i.e. region supplied by a coronary artery not infused with doxorubicin). We found no differences in CMR-quantitative perfusion or CFR between infused and remote regions after a single doxorubicin injection (*Figure 3*).

Upon histological examination 48 h and 2 weeks after a single IC doxorubicin injection, the only structural abnormality observed was a minimal to slight hyperplasia and only occasional loss of smooth muscle cells in a few number of arteries. Arterioles from doxorubicin-treated pigs did not differ from controls (*Figure 4*). Similarly, capillary density after a single IC doxorubicin dose did not differ from controls at 48 h (1320±259 capillaries/mm^2^ vs. 1470±310 in controls; *P*=0.1) or at 2 weeks (1410±227 capillaries/mm^2^ vs. 1470±310 in controls; *P*=1) (*Figure 5*). Similarly, there were no differences between infused and remote regions at these two time points (*Figures 4 and 5* and Supplementary material online, *Figure S4*). We also did not find changes in terms of CMR-based tissue characterization sequences, oedema or fibrosis in the histology (Supplementary material online, *Figure S5*).

### 3.2 Changes in microcirculation status at early stages of anthracycline-induced cardiotoxicity

In our model, from baseline to Week 6 represents a subclinical stage of cardiotoxicity, in which no LV motion abnormalities are detected, as previously reported.^11^ In this study, all groups followed the same study protocol until Week 6 (including the same anthracycline regime, with doxorubicin injections at Weeks 0, 2, and 4). Therefore, we were able to group data up to this stage from all groups for CMR-determined LVEF, CMR-determined quantitative perfusion, and invasively determined CFR. In addition, a group of animals were sacrificed at Week 6 to study histological changes as well as *ex vivo* vascular motion in explanted coronary arteries.

LVEF and regional wall thickening at 6 weeks (2 weeks after the third dose) remained unchanged from baseline (pre-doxorubicin) (*Figure 2A and B*). Echo-strain obtained in a subgroup of animals confirmed the lack of changes in myocardial tissue deformation at this early time point (*Figure 2C*). When comparing infused and remote regions, no changes in any of the cardiac motion or contractility parameters were noted (*Figure 2A–D*). In contrast, CMR-determined quantitative perfusion declined progressively and was significantly below baseline values by Week 6 (199±40 mL/100 g/min at baseline vs. 141±23.3 mL/100 g/min after 3 fortnightly doxorubicin doses, *P*=0.012) (*Figure 3A*). Invasive determination of CFR showed a similar pattern, with a progressive reduction in microcirculation function. This decline was already statistically significant at 4 weeks (3.8±1.11 at baseline vs. 2.51±0.82 after 2 fortnightly doxorubicin doses, *P*<0.001). CFR was further reduced after 6 weeks (3.8±1.11 at baseline vs. 2.51±0.71 after 3 fortnightly doxorubicin doses, *P*=0.001) (*Figure 3B*). CMR-measured perfusion and CFR in remote regions at these early stages were not statistically different from baseline (*Figure 3A and B*, bottom panels).

Histological evaluation at 6 weeks (2 weeks after the third fortnightly doxorubicin injection) revealed structural alterations in arteries of all calibres (small, medium, and large) (*Figure 4A*). These changes were mild but statistically significant vs. control arteries (*P*=0.005). Alterations included slight to moderate tunica intima hyperplasia and tunica media degeneration (*Figure 4B*). CD31 immunohistochemistry at 6 weeks revealed a non-significant decrease in capillary density (1090±344 capillaries/mm^2^ in doxorubicin-injected pigs vs. 1470±310 in controls, *P*=0.43) (*Figure 5*).

Additional structural vascular parameters in the histology (Supplementary material online, *Figure S6*) as well as in apoptosis by TUNEL and other apoptotic markers (Supplementary material online, *Figures S7 and S8*) were also numerically increased in animals receiving anthracyclines and in the long-term stage.

**Figure 6:**
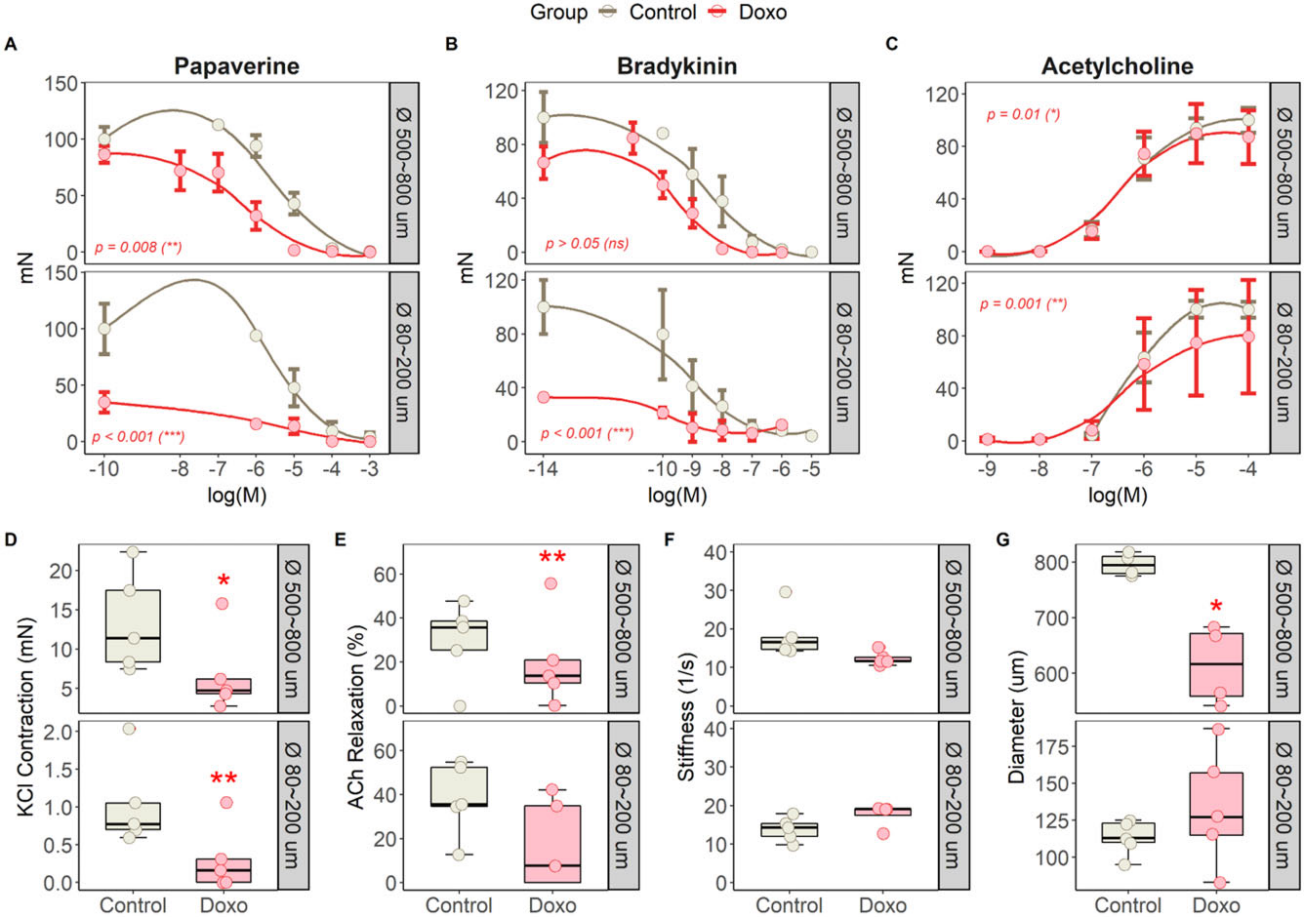
*Ex vivo* coronary artery vascular reactivity evaluation on myography during early subclinical anthracycline-induced cardiotoxicity. Absolute vascular response (in mN) after increasing doses of (*A*) papaverine, (*B*) bradykinin, and (*C*) acetylcholine. Boxplots and individual dots for (*D*) KCl contraction (mN), (*E*) ACh relaxation (%), (*F*) stiffness (1/s), and (*G*) diameter. *N*=5 animals per group. Asterisks indicate statistically significant differences between both groups: *P<0.05, **P<0.01, ***P<0.001. Differences between control and doxorubicin animal arteries subjected to several drugs [panels (*A*)–(*C*)] were detected with ANOVA analysis comparing the two multinomial logistic models. Unpaired Students’ *t*-test was performed to detect differences between both groups in panels (*D*)–(*G*).

To further study the mechanism leading to microvascular dysfunction (i.e. structural damage vs. external factors, such as circulating humoral ones) at this early stage of cardiotoxicity, we performed additional *ex vivo* studies on explanted LAD coronary arteries on myography. Surprisingly, arteries from animals receiving doxorubicin were unable to have strong and sustainable contractile responses upon stimulation with different vasoconstrictor agents (*Figure 6*). This phenomenon, probably due to a massive damage of the muscular layer, impede us to properly evaluate vasodilation capacity when using vasodilator agents, such as the smooth muscle cell-mediated vasodilator papaverine (*Figure 6A*) and the endothelium-mediated vasodilator bradykinin (*Figure 6B*). Both concentration-response curves showed an aberrant vasodilation response likely due to lower initial contraction in arteries from doxorubicin-treated animals. Interestingly, acetylcholine-induced endothelium-dependent relaxation (second wave, Supplementary material online, *Figure S3B*) was also compromised in animals receiving doxorubicin (*Figure 6E*), thus suggesting a damaged endothelium.

Vessel stiffness did not reveal any significant differences between groups (*Figure 6F*). Conversely, arterial diameters of LAD arteries from animals receiving doxorubicin until 6 weeks were smaller (*Figure 6G*).

### 3.3 Long-term changes in microcirculation status in animals receiving low cumulative anthracyclines dosing

After showing that after only three injections, microcirculation was already damaged, we wanted to explore the persistence or reversibility of the doxorubicin-induced anatomical and functional microvascular alterations in the low cumulative dose protocol (i.e. three doxorubicin injections 2 weeks apart). Animals were thus monitored by serial *in vivo* microvascular evaluations (perfusion and CFR) until Week 16 (12 weeks after the third and final IC doxorubicin injection), followed by histology evaluation at the end of the protocol (see protocol scheme on *Figure 1*).

LVEF and regional contractility were unaltered relative to baseline at 6 weeks, and remained the same at 16 weeks follow-up (53.7±4.21 at baseline vs. 57.1±4.05% at 16 weeks, *P*=0.14 for LVEF, *Figures 2A and B*). CMR-determined perfusion was reduced at 6 weeks (as described above) and remained low at 16 weeks for infused (158±19.1 vs. 122±13.4 mL/100 g/min at 6 and 16 weeks, respectively, *P*=0.63) and remote areas (146±31.5 vs. 138±11.9 mL/100 g/min at 6 and 16 weeks, respectively, *P*=0.95) (*Figure 3A*). Invasively determined CFR showed a similar pattern (3.52±0.89 vs. 3.13±0.82 at baseline and 16 weeks, respectively, *P*=0.13) (*Figure 3B*).

Histological evaluation of the low cumulative dose group at the end of the 16-week protocol showed a similar pattern to that seen at 2 weeks after third doxorubicin injection (slight to moderate tunica intima hyperplasia and tunica media degeneration) (*Figure 4*). Capillary density at 16 weeks was also similar to that observed 2 weeks after the third dose, showing non-significant reduction relative to controls (1470±310 capillaries/mm^2^ in controls vs. 1070±307 capillaries/mm^2^ at 16 weeks, *P*=0.4) (*Figure 5*). Apoptotic markers remained overexpressed (albeit not significant compared with control animals) at this late stage (Supplementary material online, *Figures S7 and S8*). In the remote myocardium, capillary density showed a similar but attenuated pattern (*Figures 4A and 5A*) than in the infused region.

Altogether, these data show that the damage to the microcirculation observed in early stages (i.e. after only three doxorubicin doses) is not reversed in the long term despite stopping the chemotherapy protocol (yielding low cumulative doxorubicin dose).

### 3.4 Long-term changes in microcirculation status in animals receiving high cumulative anthracyclines dosing

As previously reported,^11^ 5 injections of doxorubicin 2 weeks apart results in a full-blown cardiotoxicity phenotype, with massive reduction in long-term LVEF (52.5±1.5 at baseline vs. 30.4±7.7% at 16 weeks, *P*<0.001, *Figure 2A*). Here, we further characterized this phenotype by showing that at long-term (i.e. 16 weeks follow-up, 8 weeks after fifth injection), both infused and remote regions show a reduction in regional contractility: wall thickening in infused region was 70.6±19.9 at baseline vs. 18.4±20.2% at 16 weeks (*P*=0.004), and in remote region was 63.9±12.7 at baseline vs. 36.2±19.2% at 16 weeks (*P*=0.003) (*Figure 2B*).

CMR-determined perfusion, which was already reduced at 6 weeks, declined sharply at week 16 (135±4.9 vs. 65.4±18.2 mL/100 g/min at 6 and 16 weeks, respectively, *P*<0.001) (*Figure 3A*). CMR-perfusion was also altered in the remote areas at the end of the study (209±55.4 vs. 114±10 mL/100 g/min at baseline and 16 weeks, respectively, *P*=0.01). Invasive evaluation of the microcirculatory function showed a similar pattern of progressive decline in CFR, reaching its lowest value at 16 weeks follow-up (2.1±0.2 vs. 1.41±0.23 at 6 and 16 weeks, respectively, *P*=0.001) (*Figure 3B*). Invasive CRF was also altered in the remote areas at the end of the study (3.08±1.2 vs. 2.66±0.61 at baseline and 16 weeks, respectively, *P*=0.01).

Histological evaluation at 16 weeks in animal receiving high cumulative doxorubicin dosing revealed massive injury in arteries and arterioles (*P*<0.001 vs. control animals) (*Figure 4A*). Structural alterations were detected in arteries of all calibres (small, medium, and large) and in arterioles with massive loss of arteriolar density (Supplementary material online, *Figure S6*); these changes included Grade 4 damage, featuring moderate-to-severe tunica intima hyperplasia and luminal stenosis or massive smooth-muscle-cell loss in the tunica media accompanied by extracellular matrix replacement. Capillary density showed a very sharp decline at late-stage cardiotoxicity, from 1470±310 capillaries/mm^2^ at baseline to 394±271 capillaries/mm^2^ in the 16-week *‘Long-term (5 doses)’* group (*P*<0.001) (*Figure 5*). Apoptosis was up-regulated at that time with more apoptotic nuclei in the infused area from the doxorubicin-treated animals and overexpression of related-proteins (Supplementary material online, *Figures S7 and S8*).

A similar but more attenuated pattern of structural and functional microvascular deterioration was observed at 16 weeks in the remote region in the histological evaluation (*Figure 4*).

## 4. Discussion

In this study, we combined state-of-the-art techniques for monitoring myocardial perfusion (CMR^12^) and microcirculatory function (invasive CFR^15^) with quantitative histology to study the progress of microvascular damage at different stages of anthracycline-induced cardiotoxicity in pigs. Our main findings are summarized as follows (Graphical Abstract): (i) IC injection of doxorubicin (0.45 mg/kg) did not induce acute coronary microvascular injury, excluding immediate chemically induced damage secondary to the administration route. (ii) At subclinical stages (before the onset of LV motion/contratile defects), the microcirculation showed signs of progressive deterioration of anatomy (perfusion) and function (CFR). These *in vivo* defects were accompanied by mild histological alterations to arteries, including mild-to-moderate tunica hyperplasia accompanied by myofibroblast proliferation and collagen accumulation and *ex vivo* arterial vasomotion defects with a main muscular component during the early stage of the disease. (iii) The mild damage to the microcirculation detected at subclinical stages was persistent, with no reversion at the end of the protocol even when doxorubicin treatment was curtailed after three injections (i.e. low cumulative dose). (iv) A high cumulative doxorubicin dose (five fortnightly IC injections) induced manifest cardiotoxicity, with severe deterioration in LV function accompanied by massive and irreversible injury to the microcirculation at the anatomical and functional levels.

By confirming that anthracyclines irreversibly damage the myocardial microcirculation, these findings have important clinical implications. Damage to the coronary microcirculation has been consistently linked to poor cardiovascular outcomes in patients with a number of conditions,^16^^,^^17^ and anthracycline-induced injury in this vascular compartment might be an important contributor to the adverse cardiovascular outcomes seen in cancer survivors who received anthracyclines, even those who did not develop clinically manifest cardiotoxicity (LV motion abnormalities).^10^^,^^18^ There is a growing interest in the impact of cancer therapies on the vascular system.^19^^,^^20^ Most experimental evidence in this area comes from *in vitro* studies testing the vulnerability of endothelial and smooth muscle cells to cancer treatments. Endothelial-cell damage upon exposure to these agents is mediated by increased ROS production and direct DNA damage, via pathways similar to those described in cardiomyocytes.^21^^,^^22^ Like these studies, Murata *et al*.^6^ described anthracycline-induced apoptotic changes and DNA fragmentation in isolated cells, but these authors also reported functional disruption of vascular contractility with increasing anthracycline doses. Our study confirms the association between increasing cumulative doses and vascular damage in an *in vivo* setting.

A deleterious effect of anthracyclines on the microcirculation of the heart and other organs was reported in earlier *in vivo* studies in small animal models.^8^^,^^9^^,^^23^^,^^24^ Using a rat model of doxorubicin intraperitoneal injection, Eckman *et al*.^9^ demonstrated early coronary remodeling after a high cumulative dose. Huang *et al*.^8^ reported that subtle cardiotoxic microvascular impairment in juvenile mice predisposed the animals to rapid-onset heart failure in adulthood and overt cardiotoxicity upon exercise training. Ours is the first large-animal study to explore the impact of doxorubicin treatment on the coronary microcirculation. We chose the pig because of its closeness to human cardiac anatomy and physiology and its compatibility with tools used clinically to assess coronary microcirculation: CMR and invasive coronary catheters. This amenability to serial *in vivo* assessments is a key advantage of our model, allowing study of the entire cardiotoxic process from its subclinical to manifest stages in a model close to humans.

Vascular injury associated with anti-cancer interventions (including anthracyclines) has been studied extensively in the clinical setting,^10^^,^^25^^,^^26^ and cardiac perfusion has been proposed previously as a potential tool for studying the adverse effects of chemotherapy on the heart.^18^ Using single-photon emission computed tomography (SPECT), Hardenbergh *et al*.^25^ found visible perfusion defects in ∼60% of breast-cancer patients treated with radiotherapy alone, but the figure rose to 100% for patients receiving radiotherapy in combination with doxorubicin. Gallucci *et al*.^10^ used stress-SPECT to expose perfusion defects in breast-cancer survivors. They found that 23.3% of the enrolled patients had some grade of myocardial perfusion defect. In this study, we combined CMR-based quantitative myocardial perfusion at rest with invasive assessment of coronary physiology, providing a comprehensive *in vivo* evaluation of microcirculation status from the structural and functional perspectives. Consistent with previous human studies, we found that microvascular injury was not limited to animals that developed LV systolic dysfunction and that even subclinical cardiotoxicity stages were associated with irreversible microcirculation damage. The acute impact of a single doxorubicin dose was previously tested by Laursen *et al*.^27^ who imaged myocardial perfusion in 70 lymphoma patients by rest/stress Rubidium^82^ positron emission tomography before and after their first doxorubicin exposure; the authors found defects in myocardial perfusion reserve and in myocardial perfusion during stress but not at rest. Opposed to this, we found no acute myocardial perfusion defects or CFR alterations after a single IC doxorubicin dose, but we did observe mild structural damage at 48 h and 2 weeks after a single injection.

Our study thus shows that the coronary microvasculature can be significantly and irreversibly damaged by anthracycline treatment even when no clinically overt cardiotoxicity is observed (no LV motion defects). These findings may partially explain the high long-term risk of cancer survivors for adverse cardiovascular outcomes. Given that microcirculation status can be assessed non-invasively by CMR,^12^ this modality would be an attractive strategy for stratifying cardiovascular risk in cancer survivors who received anthracyclines. In addition, quantitative perfusion CMR is sensible to the effects in all vascular territories (arteries and arterioles) being more sensible that invasive CFR that it is intended to study the vascular bed.

## 5. Conclusions

Serial *in vivo* evaluations of microcirculation status using state-of-the-art CMR and CFR showed that serial exposure to anthracyclines is associated with progressive and irreversible damage to the microcirculation. This long-persisting damage is apparent from the early stages of anthracycline exposure. Microcirculation damage might explain some of the increased incidence of cardiovascular events in cancer survivors who received anthracyclines without showing cardiac contractile defects.

### 5.1 Study limitations

Doxorubicin IC route of administration is not ideal due to its potential direct effect on the coronary microvasculature and the lack of systemic inflammatory effect that i.v. doxorubicin administration may be produced in patients, potentially contributing to cardiotoxicity. The modified protocol used here^28^^,^^29^ was selected because the myelosuppressive effects of i.v. doxorubicin are pronounced in pigs,^30^ compromising the experimental setting. To exclude a direct chemical effect, we included two acute groups in the model (48 h and 2 weeks after 1 dose of IC doxorubicin). These treatments revealed an insignificant short-term impact of doxorubicin exposure on cardiac perfusion and CFR, demonstrating that the cardiotoxic effect on the coronary vasculature is secondary to cumulative dosing. There is a lack of functional assessment of conduit arteries and the absence of LVEF measurements under increased load stress conditions. Our study did not include measurements of flow during pharmacological vasodilation at the point where flow reserve is depressed to enhance the interpretation of the flow reserve measurements and the lack of haemodynamic records during papaverine infusion. We did not collect blood from the coronary sinus to study changes in pO2 during cardiotoxicity development. In the myograph experiments, endothelial dilator function could not be completely evaluated due to the presence of an already injured smooth muscle cell layer. Finally, we only used castrated male pigs in this study to reduce the variability that a sex and hormones may introduce in results.

## Supplementary material

Supplementary material is available at *Cardiovascular Research* online.

## Authors’ contributions

C.G.-A.: drafting the work, acquisition, and interpretation of data. J.P.V.-T.: drafting the work, acquisition, and interpretation of data. M.L.: critical revision of the manuscript and interpretation of data. G.J.L.: acquisition of data. A.d.M.-I.: interpretation of data. C.P.-M.: acquisition of data. Á.M.: myograph experiments. I.A.D.-R.: acquisition of data. R.V.-G.: apoptosis analyses and critical revision of the manuscript. E.O.: critical revision of the manuscript. V.F.: critical revision of the manuscript. J.S.-G.: conception and design of the work and final revision of the manuscript. B.I.: conception and design of the work and final revision of the manuscript. Takes full responsibility for the study. All authors approved the final version of the manuscript.

**Conflict of interest**

J.S.-G. is employed by Philips Healthcare. All other authors have no conflict to declare.

## Funding

This study was supported by a European Research Council grant MATRIX (ERC-COG-2018-ID: 819775) to B.I.; the ERA-CVD Joint Translational Call 2016 [funded through the Instituto de Salud Carlos III (ISCIII), and the European Regional Development Fund (ERDF); ID: AC16/00021]; a Health Research Project from the ISCIII-FIS (ID: PI16/02110); The BBVA foundation grant (ID: BIO CAR 0265). This study forms part of a Master Research Agreement between the CNIC and Philips Healthcare. C.G. and R.V.-G. are P-FIS fellows (Instituto de Salud Carlos III). E.O. is recipient of funds from the Programa de Atracción de Talento (2017-T1/BMD-5185) of the Comunidad de Madrid. This study was partially supported by the Comunidad de Madrid (S2017/BMD-3867 RENIM-CM) and cofunded with European Union structural and investment funds. The CNIC was supported by the Instituto de Salud Carlos III (ISCIII), the Ministerio de Ciencia, Innovación y Universidades (MCNU), and the Pro CNIC Foundation.

## Data availability

The data underlying this article will be shared on reasonable request to the corresponding author.

## Supplementary Material

cvab053_Supplementary_Data

## Notes

Translational perspective By using a highly translatable large-animal model, this work shows that cumulative exposure to anthracycline results in an irreversible damage to the heart microcirculation. The microcirculation injury is observed even before overt left ventricular cardiac systolic dysfunction (surrogate for overt cardiotoxicity) is present. Cardiac microcirculation damage is associated with poor clinical outcome in general, and thus the present findings might partially explain the high incidence of cardiovascular adverse outcomes in cancer survivors even in the absence of overt cardiotoxicity.
