# Supplementary material for: Coronary microcirculation damage in anthracycline cardiotoxicity
Source: Cardiovasc Res. 2021 Feb 19;118(2):531–41. doi: 10.1093/cvr/cvab053 (PMC8803079; doi:10.1093/cvr/cvab053)
Supplement: cvab053_Supplementary_Data [file cvab053_supplementary_data.pdf]

Coronary microcirculation damage in anthracycline cardiotoxicity

Supplementary material

Echocardiographic exam

Echocardiography was done in the myograph ExPr. 3 group at baseline at before each doxorubicin infusion at 2 and 4 weeks as well as before sacrifice at week 6. These exams were performed by the same researcher using an iE33 ultrasound system (Philips Healthcare, USA) equipped with a transthoracic probe X5-1 in all the timepoints and animals. Exams consisted in a left parasternal short axis view at mitral valve, papillary muscles and apical levels in order to assess circumferential strain analyses.

All studies were analyzes by a blind observer using specific ultrasound software by Philips. Strain parameters were obtained for the three views. For analyzing changes in infused and remote areas, segments 7 and 8 and segments 10 and 11 in the medium axis were averaged for infused and remote respectively.

Left Atrium Reservoir

LA volumes and function were defined as follows. Because no volumetric estimation methods have been validated in pigs, we quantified LA dimensions based on the mean area from the 4-chamber view. From this view, a phasic parameter were derived as follows: Reservoir function or expansion index (%): 100 · (maximal LA area − minimum LA area)/minimum LA area

TUNEL protocol

Double-strand DNA breaks generated during apoptosis was assessed using a green fluorescence Abbkine TUNEL Apoptosis Detection Kit. Briefly, after dewax and rehydrate, paraffin tissue sections were incubated with a mixture containing the TdT enzyme and incubated for 60 min at 37ºC in a dark chamber. After washing, sections were counterstained with DAPI and visualized with a Leica SP8 confocal microscope.

Western Blot

Heart tissue from infused area was lysed in RIPA buffer supplemented with a protease and phosphatase inhibitor cocktail. Protein content was quantified with the Bio-Rad BCA protein assay. Protein samples were separated by SDS–polyacrylamide gel electrophoresis, and proteins were transferred to nitrocellulose membranes. After blocking, membranes were incubated with the following antibodies: p53 (Affinity Biosciences, AF0877), BCL2 (Elabscience, E-AB-22004), BAX (Boster biological, M00183-1) and GAPDH (Abcam, ab8245) at 4°C overnight. Bound antibodies were detected after staining with a corresponding secondary antibody. Quantitative densitometric analysis was performed using ImageJ Fiji software.

Supplementary Figures

Supplementary Figure 1. Representative Masson's trichrome histological images for each "Damage Score" for arteries (small, medium and large size) and arterioles

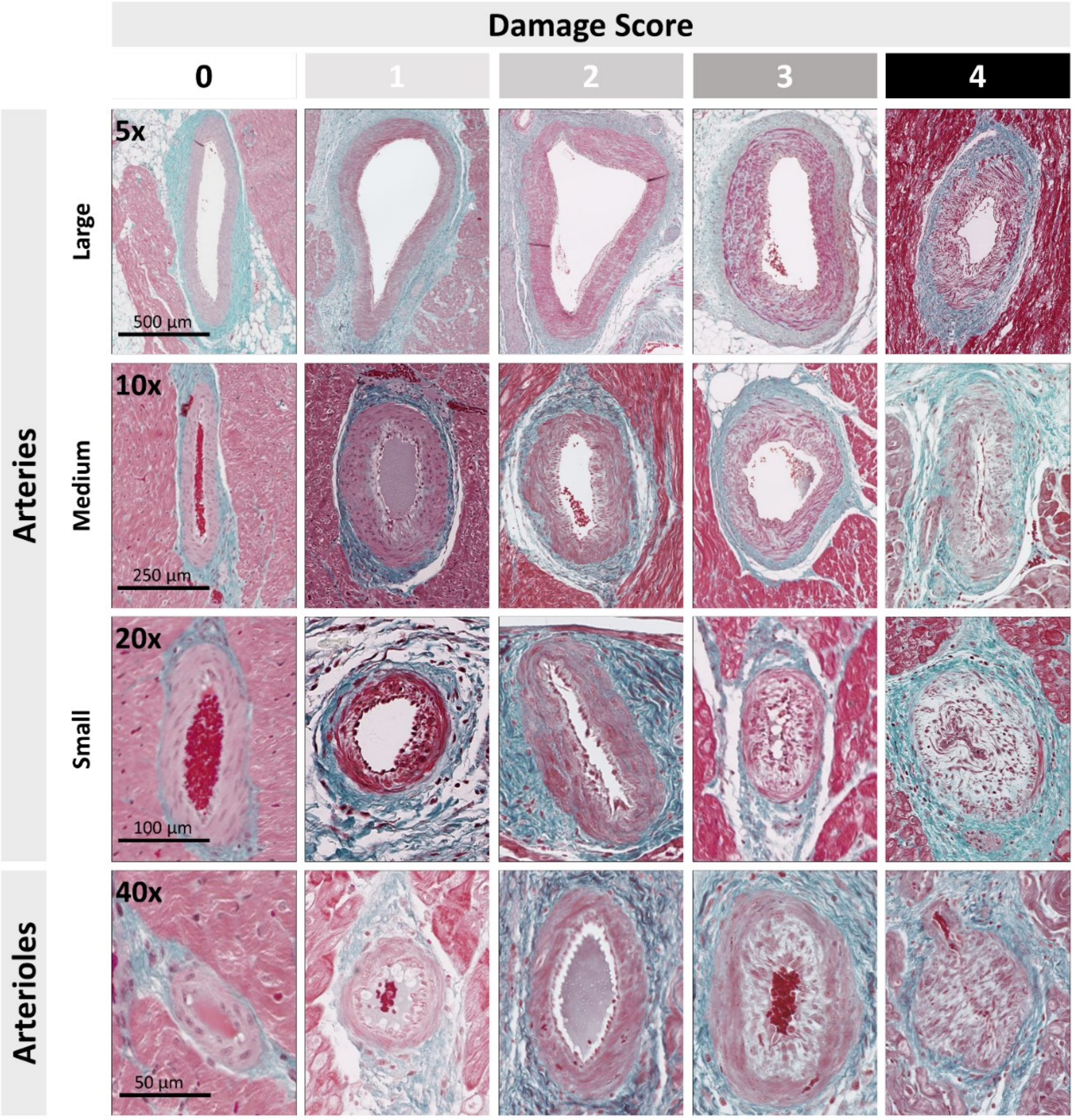

Supplementary Figure 2. Scheme of the ImageJ macros for CD31, SMA and fibrosis quantification

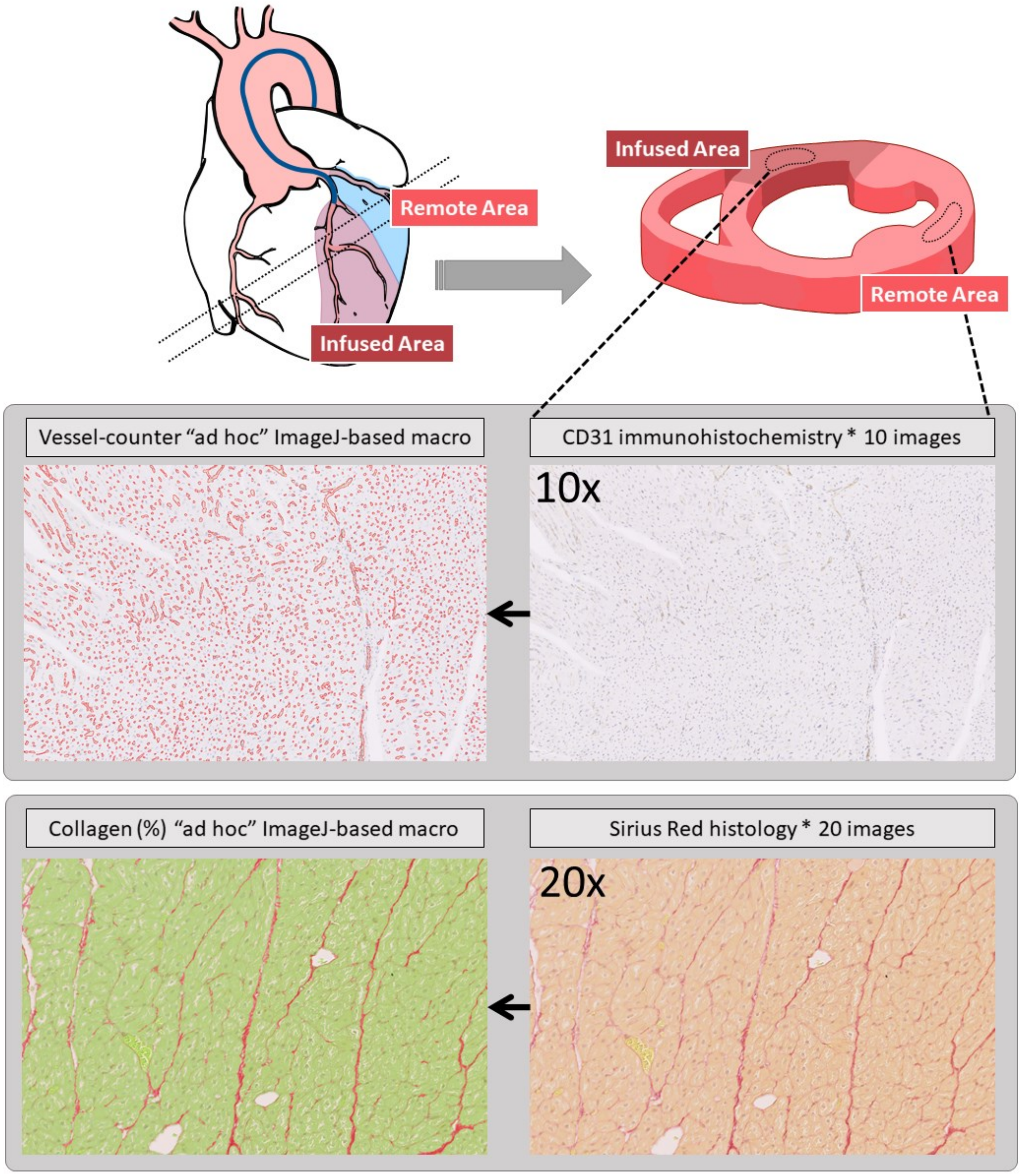

10 images of 10x magnification were obtained from CD31 immunohistochemistry from the doxorubicin infused area in each animal from the study. The macro marks and counts in red each capillary in the sample for CD31, obtaining de density in the tissue area. Regarding fibrosis quantification, 20 images of 20x magnification were obtained from Sirius Red Staining.

Supplementary Figure 3. Myograph experiments

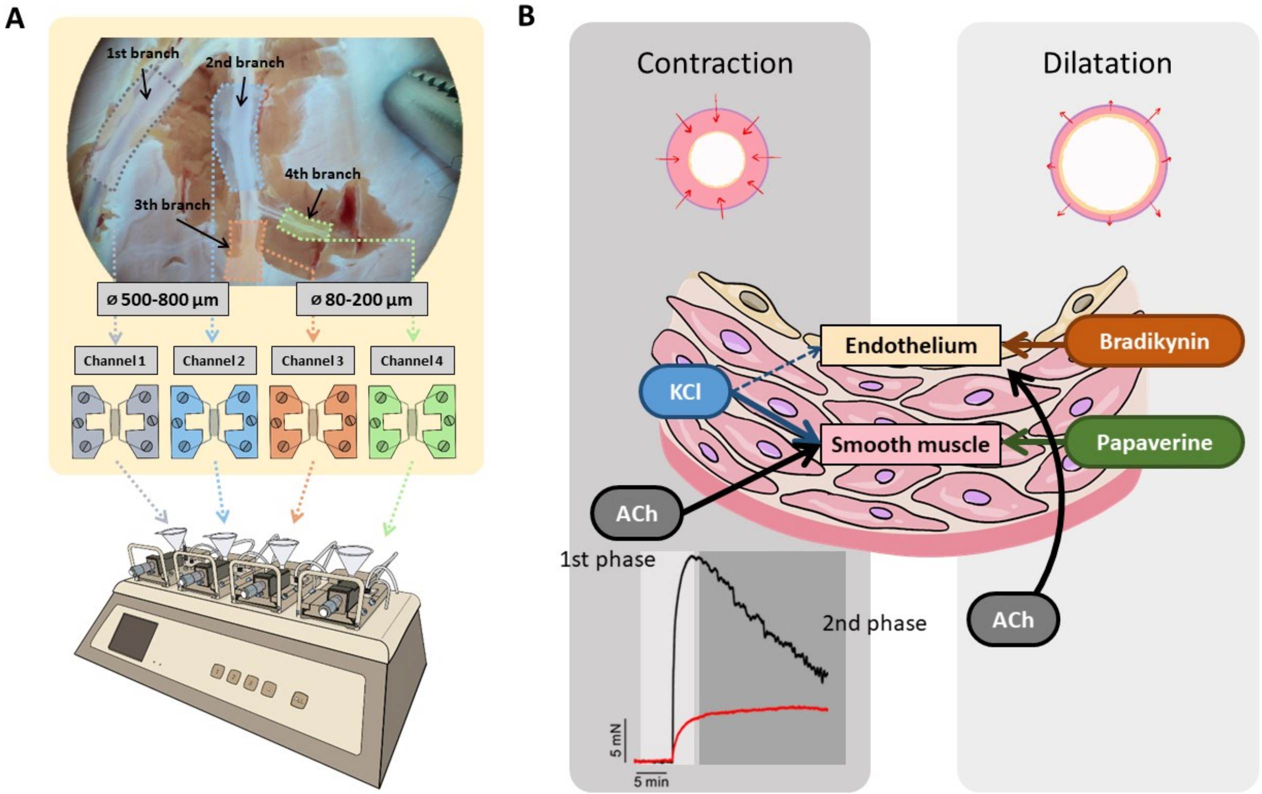

(**A**) Dissection and division of different coronary branches in the infused area and its emplacement in the myograph. (**B**) Pharmacological scheme of the different drugs and its effect used during the protocol. Early constriction and late dilation waves of ACh are indicated with an example of control (line plot in black) and doxo groups (line plot in red). The dual antithetical effects of Ach on the coronary arteries (early constriction and late dilation) is explained. among others. in a previous article by Radico et al[1].

Supplementary Figure 4. Hemodynamic parameters from the early group performed for the myograph study

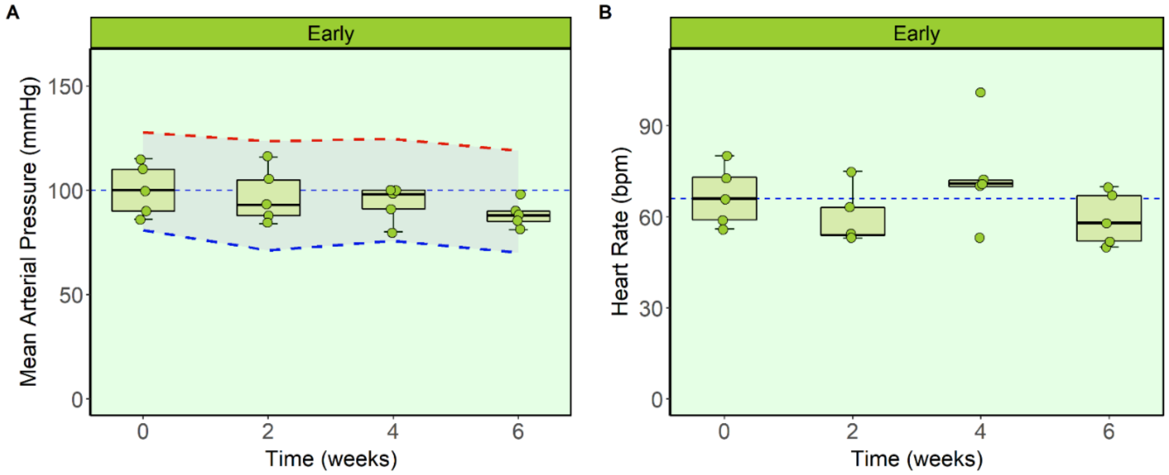

(**A**) Boxplots and individual data for mean arterial pressure over time (dashed lines represent maximum and minimum AP) and (**B**) Heart rate (bpm). One-way paired ANOVA (corrected by Bonferroni) was performed to identify statistical differences between timepoints. N=5. Asterisks indicate statistically significant differences compared with Baseline: * $p < 0.05$, ** $p < 0.01$, *** $p < 0.001$.

Supplementary Figure 5. *In vivo* and *ex vivo* tissue characterization of the ExPr. 1 and 2 groups (48h and 2 weeks)

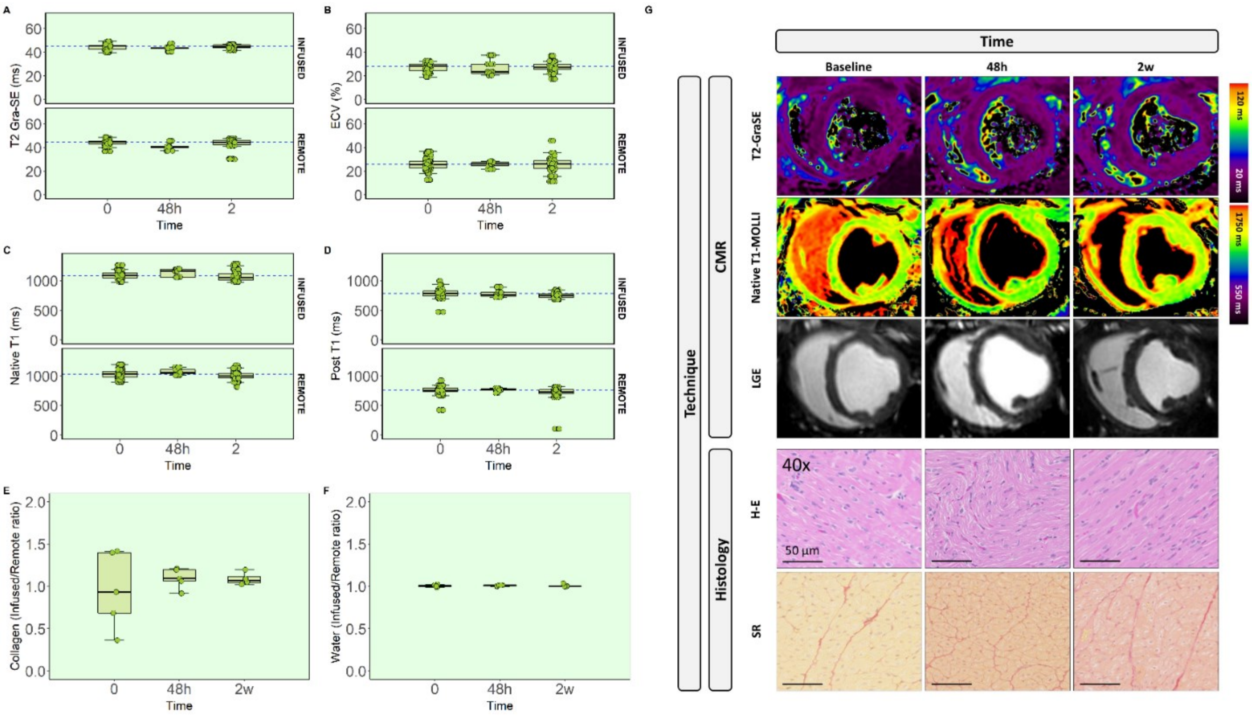

Boxplot and dotted individual data for each point divided by area of infusion for (**A**) T2-GraSE, (**B**) ECV, (**C**) T1 native and (**D**) T1 post-contrast. . Boxplots and dotted individual data for each group for (**E**) collagen fraction area and (**F**) water content of the infused zone (ration with remote values). (**G**) Representative CMR images at short axis for T2-GraSE, T1-MOLLI and Late Gadolinium Enhancement at different timepoints and histology in each group. Two-way paired ANOVA (corrected by Bonferroni; panels in B) and one-way unpaired ANOVA (panels in D) analysis were performed to identify statistical differences over time, area of infusion and experimental groups. N = 5 animals per group. Asterisks indicate statistically significant differences compared with Baseline: * $p < 0.05$, ** $p < 0.01$, *** $p < 0.001$. Hashes indicate statistically significant differences compared with respective remote: # $p < 0.05$, ## $p < 0.01$, ### $p < 0.001$.

Supplementary Figure 6. Additional vascularity analyses

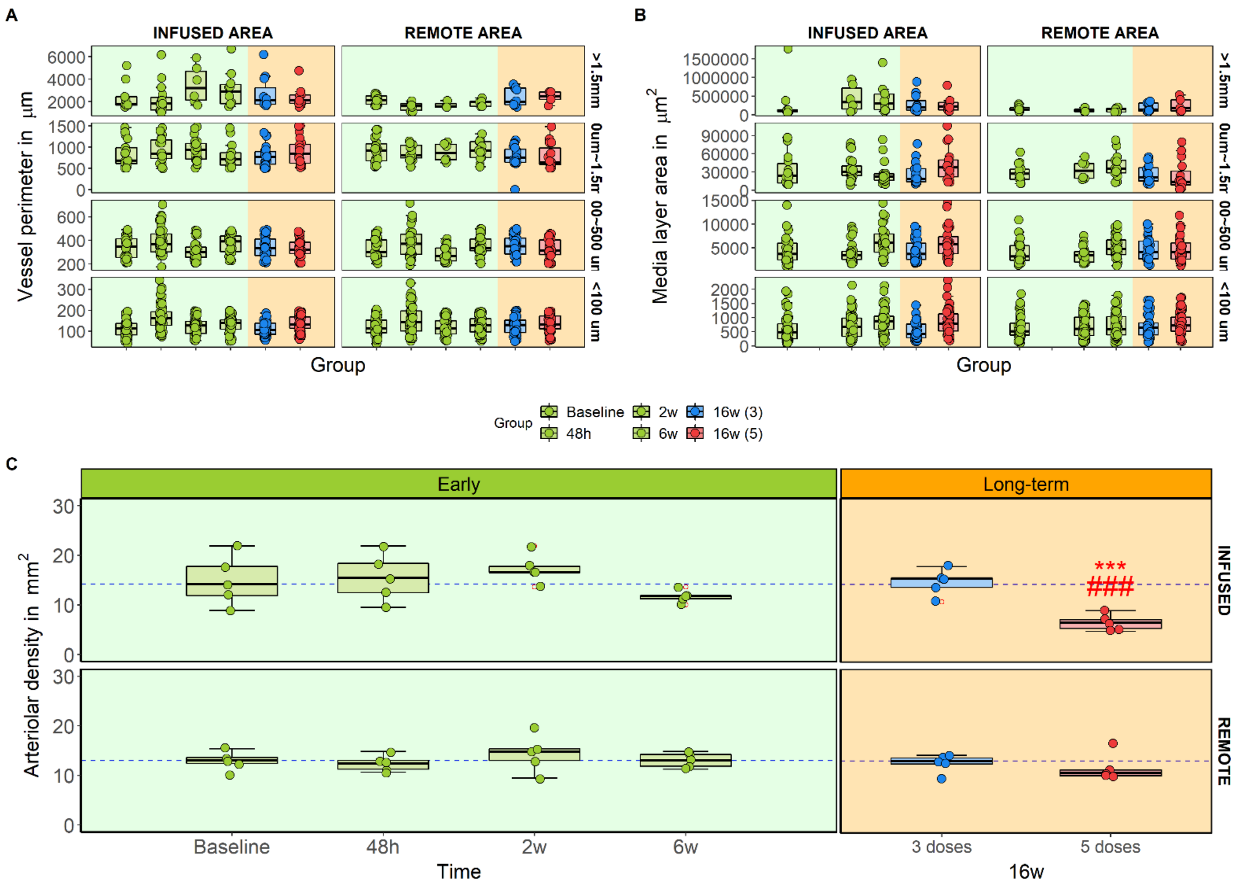

Boxplot and dotted individual vessel data for (**A**) Vessel perimeter and (**B**) Media layer area, for each artery size / arteriole and group in the remote and the infused areas. Boxplot and dotted individual data for (**C**) Arteriolar density quantification for early and chronic groups in the remote and the infused areas. Three-way paired ANOVA (corrected by Bonferroni in A and B) and two-way paired ANOVA (corrected by Bonferroni; C) analysis were performed to identify statistical differences over artery size, area of infusion and/or experimental groups. n = 5 animals, ~11 arteries measured per individual in panels A and B. Asterisks indicate statistically significant differences compared with Baseline: * $p < 0.05$, ** $p < 0.01$, *** $p < 0.001$. Hashes indicate statistically significant differences compared with respective remote: # $p < 0.05$, ## $p < 0.01$, ### $p < 0.001$.

Supplementary Figure 7. Apoptosis analyses in early and chronic groups

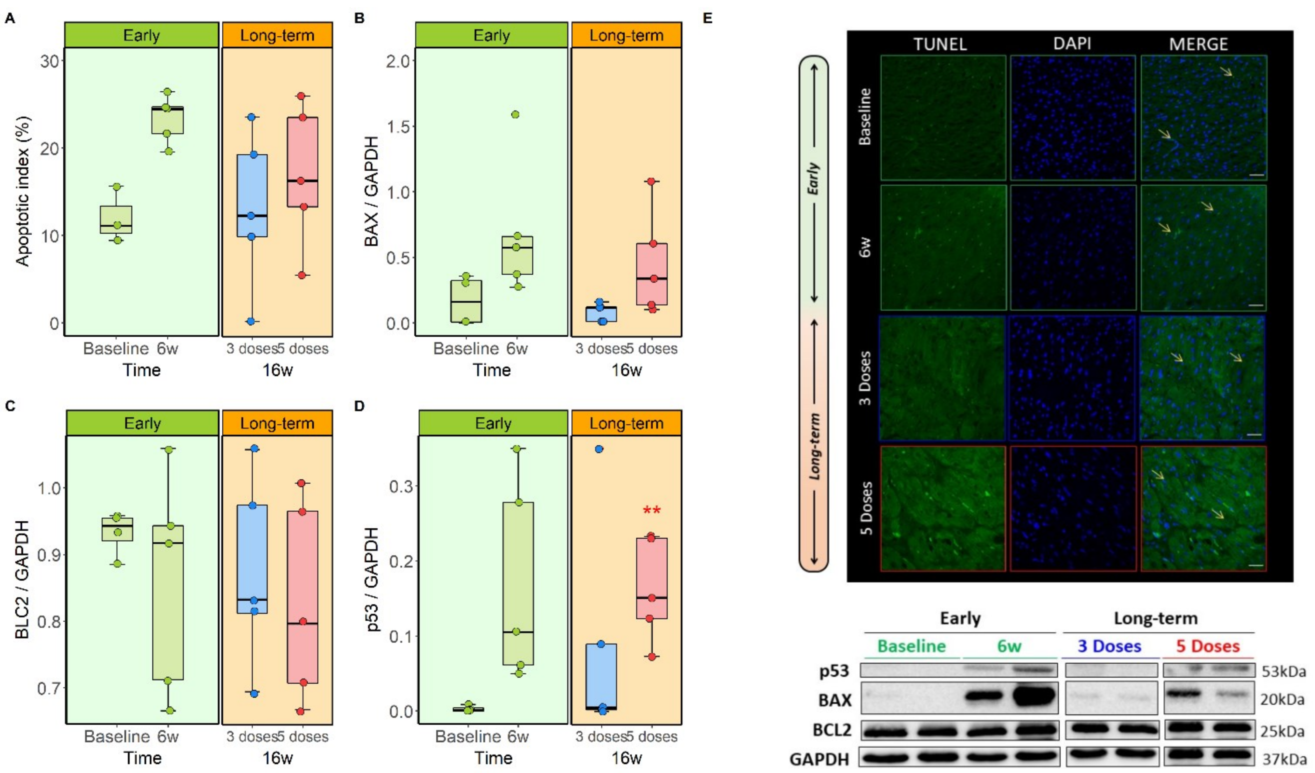

Boxplot and dotted individual data for (**A**) apoptotic index in TUNEL assessment, (**B**) BAX/GAPDH, (**C**) BLC2/GAPDH and (**D**) p53/GAPDH western blot quantifications. (**E**) Representative TUNEL, DAPI and merged images per group (top) and representative western blot bands for each group, protein of interest and GAPDH (bottom). One-way unpaired ANOVA analysis was performed to identify statistical differences between experimental groups. N = 5 (3 in the control group for TUNEL analysis and 4 in WB). Asterisks indicate statistically significant differences compared with Baseline: * $p < 0.05$, ** $p < 0.01$, *** $p < 0.001$.

Supplementary Figure 8. Western blot gels

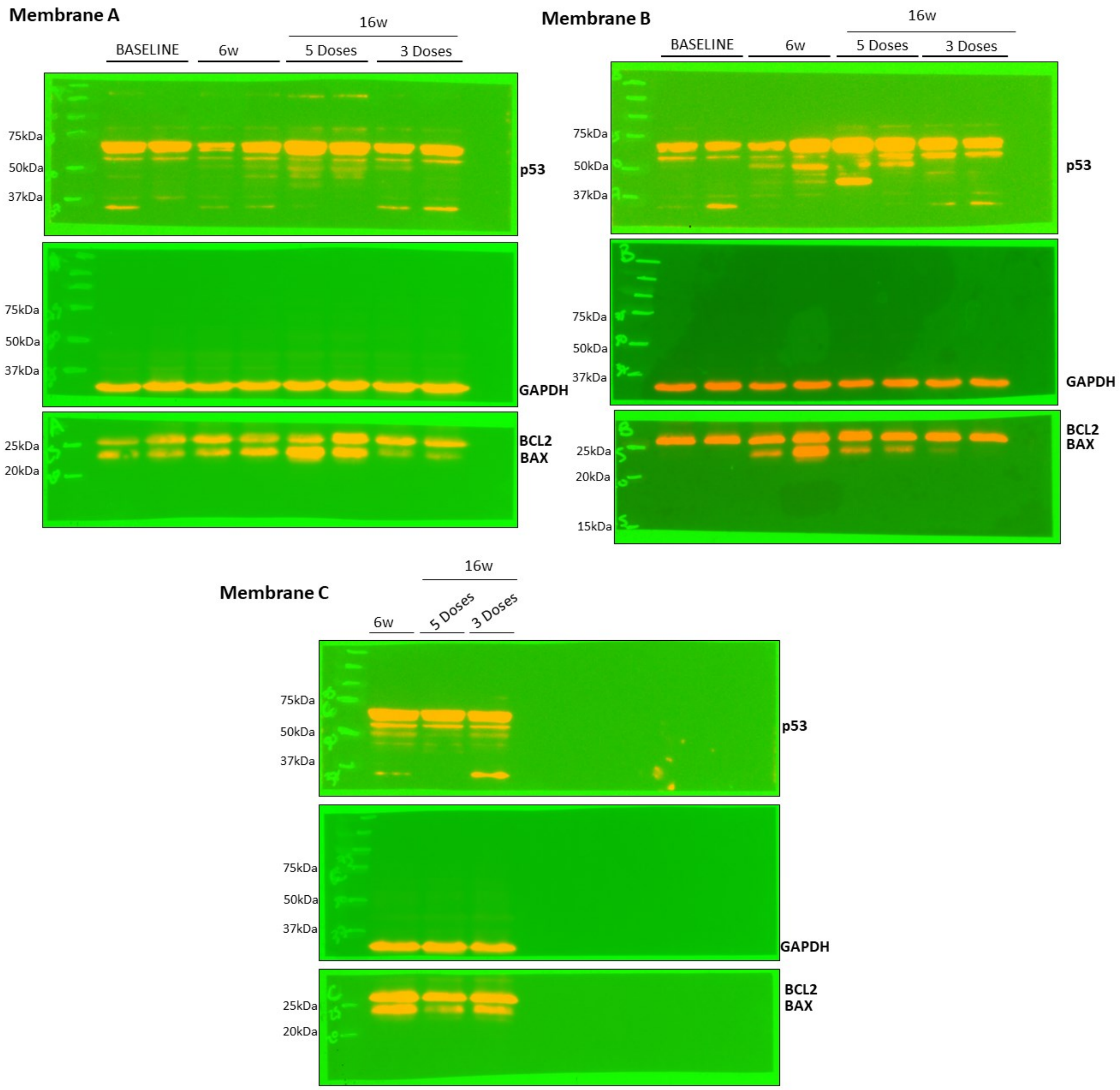

Original WB gels (divided by membranes) for the WB analysis showed in Supplementary Figure 5.

Supplementary References

1. Radico F, Cicchitti V, Zimarino M, De Caterina R. Angina pectoris and myocardial ischemia in the absence of obstructive coronary artery disease: practical considerations for diagnostic tests. *JACC Cardiovasc Interv* 2014;**7**:453-463.
